# Copper(II) Phenanthroline-Based Complexes as Potential AntiCancer Drugs: A Walkthrough on the Mechanisms of Action

**DOI:** 10.3390/molecules27010049

**Published:** 2021-12-22

**Authors:** Sebastiano Masuri, Petr Vaňhara, Maria Grazia Cabiddu, Lukáš Moráň, Josef Havel, Enzo Cadoni, Tiziana Pivetta

**Affiliations:** 1 Department of Chemical and Geological Sciences, University of Cagliari, 09042 Cagliari, Italy; mgcabidd@unica.it (M.G.C.); ecadoni@unica.it (E.C.); tpivetta@unica.it (T.P.); 2 Department of Histology and Embryology, Faculty of Medicine, Masaryk University, 62500 Brno, Czech Republic; 408080@muni.cz; 3 International Clinical Research Center, St. Anne’s University Hospital, 65691 Brno, Czech Republic; havel@chemi.muni.cz; 4 Research Centre for Applied Molecular Oncology, Masaryk Memorial Cancer Institute, 65653 Brno, Czech Republic; 5 Department of Chemistry, Faculty of Science, Masaryk University, 62500 Brno, Czech Republic

**Keywords:** cancer, coordination compounds, copper, 1,10-phenanthroline, anticancer chemotherapy, chemoresistance, cell stress response

## Abstract

Copper is an endogenous metal ion that has been studied to prepare a new antitumoral agent with less side-effects. Copper is involved as a cofactor in several enzymes, in ROS production, in the promotion of tumor progression, metastasis, and angiogenesis, and has been found at high levels in serum and tissues of several types of human cancers. Under these circumstances, two strategies are commonly followed in the development of novel anticancer Copper-based drugs: the sequestration of free Copper ions and the synthesis of Copper complexes that trigger cell death. The latter strategy has been followed in the last 40 years and many reviews have covered the anticancer properties of a broad spectrum of Copper complexes, showing that the activity of these compounds is often multi factored. In this work, we would like to focus on the anticancer properties of mixed Cu(II) complexes bearing substituted or unsubstituted 1,10-phenanthroline based ligands and different classes of inorganic and organic auxiliary ligands. For each metal complex, information regarding the tested cell lines and the mechanistic studies will be reported and discussed. The exerted action mechanisms were presented according to the auxiliary ligand/s, the metallic centers, and the increasing complexity of the compound structures.

## 1. Introduction

**Figure 1:**
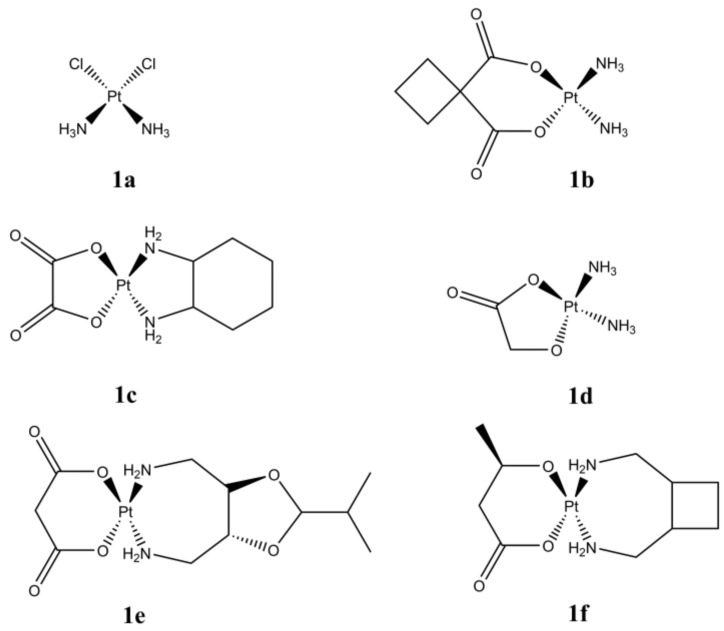
Structures of cisplatin (**a**), carboplatin (**b**), oxaliplatin (**c**), nedaplatin (**d**), heptaplatin (**e**), lobaplatin (**f**).

The fortuitous discovery of the anticancer properties of cisplatin (Figure 1a) has represented a milestone in the chemotherapeutic treatment of several types of tumors (ovarian, testicular and lung, among others) [1]. Cisplatin and its derivatives (carboplatin and oxaliplatin, Figure 1b,c) are currently approved for clinical use worldwide, while other Pt(II) anticancer metallodrugs (nedaplatin, heptaplatin, and lobaplatin Figure 1d–f) have been introduced only in a few countries [2].

As commonly known, these compounds exhibit numerous dose-dependent side effects (hepatotoxicity, nephrotoxicity, ototoxicity among the most severe). Moreover, their therapeutic efficacy is limited by pharmacoresistance phenomena that might take place after some treatment cycles [3,4].

Considering the high incidence of cancer worldwide (19.3 million new cases and 10 million deaths estimated in 2020) [5], the development of novel anticancer chemotherapeutics able to overcome the limitations of Pt(II) compounds, appears extremely important.

A common approach exploited in the design of novel anticancer metallodrugs consists in substituting Pt(II) with endogenous metal ions, with the aim of interfering at DNA level through alternative mechanisms compared to Pt(II)-based drugs (metalation), or by targeting completely different biological molecules and pathways. Moreover, the systemic toxicity of these compounds might be reduced exploiting the molecular machinery in charge of the transport and homeostasis of endogenous metal ions in biological systems [6].

Copper is an example of endogenous metal that have been studied under this perspective. It mainly serves as cofactor in several enzymes involved in different biochemical processes, such as energy production (cytochrome-c oxidase), oxidative damage prevention (superoxide dismutase), melanin production (tyrosinase) and blood coagulation (V and VIII coagulation factors). Copper absorption primarily takes place in the stomach and small intestine after being reduced as Cu(I) by ascorbate and cellular metalloreductases [7]. The distribution through the bloodstream is guaranteed by ceruloplasmin, albumin and transcuprein [8]. Cellular uptake of this metal ion occurs through the hCtr1 transport protein, which is also exploited by Pt(II)-based drugs for the same purpose [2]. Copper homeostasis in cells, tissues and organs is controlled through its blood transporters and cellular chaperones (e.g., GSH, ATOX-1, COX17 and CCS1), since an excess of free Copper ions can be potentially dangerous for human organism. It is believed that the toxicity of free Copper arises from its participation in ROS production, which is related to the accessibility of both Copper oxidation states (Cu(II) and Cu(I)) in biological conditions [9,10]. Excess of free Copper ions are correlated with the pathogenesis of Wilson disease, but it is also believed to promote tumor progression, metastasis, and angiogenesis [11]. In addition, high levels of Copper were found in serum and tissues of several types of human cancers [12,13].

Based on these findings, two different strategies are commonly followed in the development of novel anticancer Copper-based drugs: (1) sequestration of free Copper ions in excess by using metal chelators; (2) synthesis of Copper complexes that trigger cell death through accumulation of the metal ion and induction of ROS production [14].

The second strategy has been extensively followed in the last 40 years, since the discovery of Sigman et al. that the [Cu(phen)_2_]^+^ complex is able to damage DNA oxidatively, thus acting as an artificial nuclease [15]. These results have been successfully exploited in the design of many DNA-targeting metals (e.g., Ruthenium, Rhodium) complexes bearing phen-based ligands [16,17].

Many reviews have covered the anticancer properties of a broad spectrum of Copper complexes, showing how the activity of these compounds is multi factored (e.g., proteasome, topo-isomerase inhibition, induction of the apoptosis, etc.) [6,18,19,20,21,22,23].

In this work, we would like to focus on the anticancer properties of mixed Cu(II) complexes bearing 1,10-phenanthroline (phen) or substituted phenanthrolines (phen-based ligands) and different classes of inorganic (e.g., Cl-, Br-, H_2_O) and organic auxiliary ligands (e.g., carboxylic acids, α-amino acids, imines, etc.). The choice to highlight on Cu(II) complexes comes from the higher solubility, coordination numbers (typically 5 and 6) and geometries (square pyramidal, bipyramid trigonal, octahedral, all of them with different degrees of distortion) that Copper in this oxidation state can offer. The complexes here reported will be presented according to: (1) the auxiliary ligand (e.g., inorganic ligands, organic –S donor ligands, -N donor ligands); (2) number of Cu(II) centers; (3) increasing complexity in the structures of the compounds. For each metal complex, information regarding the tested cell lines and the mechanistic studies will be reported and discussed.

## 2. Mixed Cu(II) Phen-Based Complexes

### 2.1. Inorganic Auxiliary Ligands

**Figure 2:**
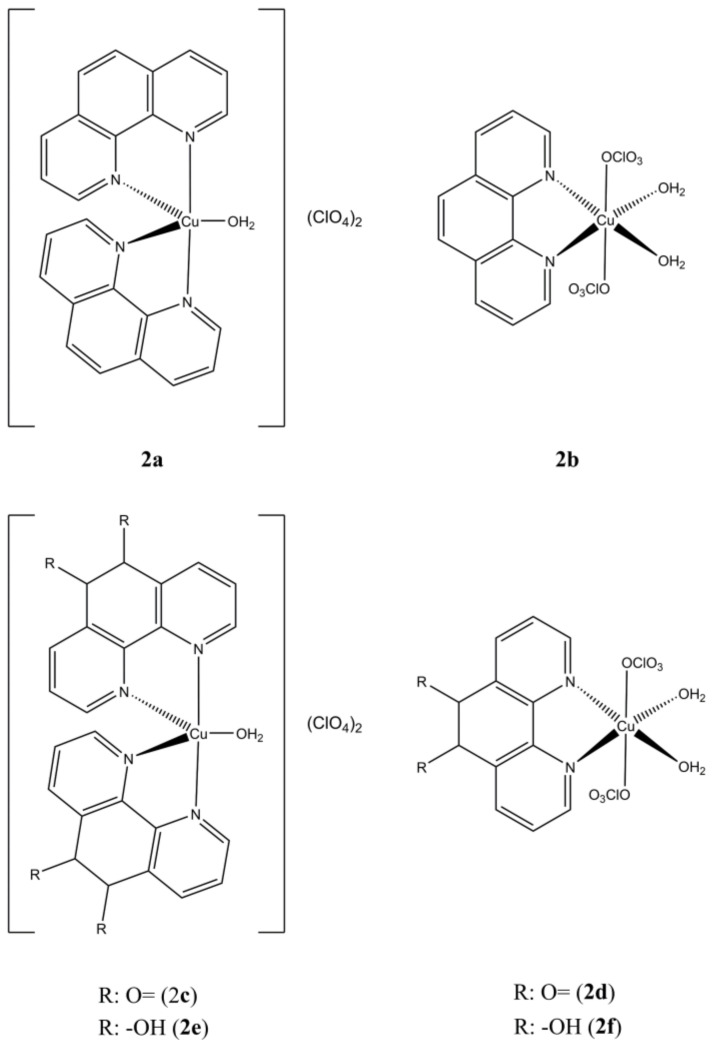
Structures of the Cu(N-N^1^)_x_(OH_2_)_y_(ClO_4_)_z_ complexes.

Mixed Cu(II) phenanthroline-based complexes having general formula Cu(N-N^1^)_x_(OH_2_)_y_(ClO_4_)_z_ (Figure 2), where “N-N^1^” are phen and some 5,6-disubstituted derivatives, were prepared and tested on a panel of solid and haematological cancer cells [24,25,26].

All the studied complexes show IC_50_ in the micro/sub-micromolar range, with complexes having two N-N^1^ units that appeared more potent compared to complexes having only one N-N^1^ unit. The potential selectivity, calculated taking the normal fibroblasts as reference, varies from a minimum 0.78 to a maximum of 19.2 according to the type and number of the phen-based moieties and the cancer cell line considered. All the studied compounds can interact with DNA, but the inversely correlated relation between DNA binding constants and anticancer potencies, brings to exclude that the biological properties observed would arise from a direct interaction with this target [25]. Recent results have partially unveiled the biological mechanism in ovarian (A-2780) cells of **2a**, which induces Endoplasmic Reticulum (ER) Stress by activating the pro-apoptotic branch of the Unfolded Protein Response (UPR), as observed by overexpression of typical biomarkers, such as PERK, IRE1 and DDIT3, and alleviation of cytotoxicity in co-administration with ER-stress modulator Taursodeoxycholic Acid (TUDCA) [27].

**Figure 3:**
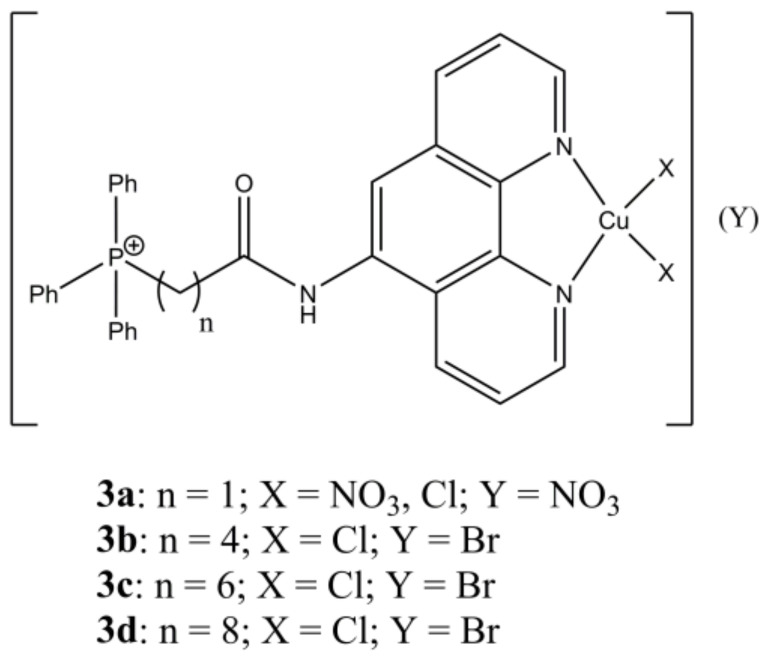
Structures of the [Cu(LPTn)_x_(X)_y_](Y)_z_ complexes.

Shi et al. have synthesized a panel of mixed complexes having general formula [Cu(LPTn)_x_(X)_y_](Y)_z_ (Figure 3), where LPTn are phenanthroline derivatives having alkyl chains of different lengths.

Both cellular uptake and activity on cervical (Hela) and ovarian (SKOV-3) cancer cells appear directly correlated with the lipophilicity of the LPTn ligand, while all of them showed mild toxicity towards healthy HK-2 cells. Intracellular ROS production and lipophilicity show a different correlation depending on the cell line, suggesting that the redox properties are not the determinant factors in the cell death induction. The most promising complex of the series (**3d**) has shown to possess anti metastatic activity, via inhibition of MMP-2 expression, in SKOV-3 cells. In addition, it also possesses antiangiogenic activity, via downregulation of VEGFR-1 expression, blocking of sprouts and tubes formation, in HUV-EC-C cells [28].

Table 1 summarizes the IC_50_ values reported for the compounds so far discussed.

### 2.2. Organic-S Donating Auxiliary Ligands

**Figure 4:**
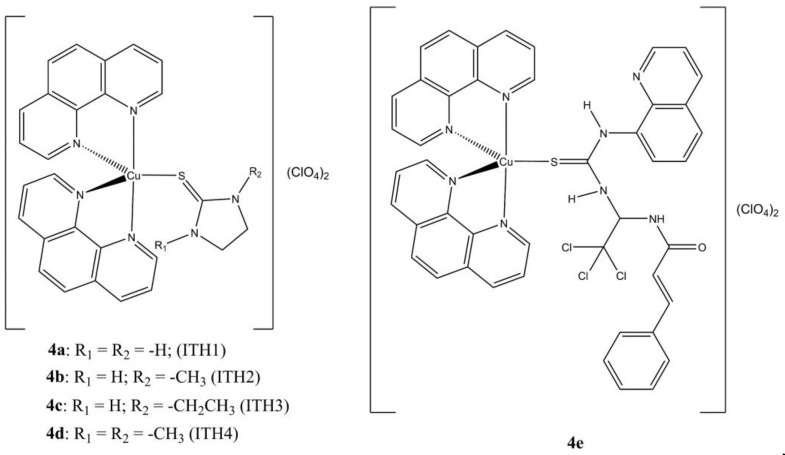
Structures of the [Cu(phen)_2_(ITHn)](ClO_4_)_2_ (**a**–**d**) and [Cu(phen)_2_(SAL)](ClO_4_)_2_ (**e**) complexes.

A series of mixed Cu(II) phenanthroline complexes of general formula [Cu(phen)_2_(ITHn)](ClO_4_)_2_, where ITHn are imidazolidine-2-thione and some of its *N*-alkylated derivatives (Figure 4a–d), have been prepared and evaluated in vitro [24]. The tested compounds show anticancer potencies in the sub-micromolar range, while the ITHn ligands are inactive (IC_50_ > 100 µM) towards the same cell lines.

As shown for **2a**, complexes **4a**–**c** exert their anticancer properties in A-2780 cells by inducing ER-stress and activating the pro-apoptotic branch of UPR. The cytotoxicity of these compounds, which differs according to the alkyl groups in the ITHn backbone, can be reduced by co-administration with TUDCA [27].

Since the ITH1 structure resembles the one of ER-stress modulator Salubrinal (SAL), the same authors explored the possibility of a chemical reactivity between SAL and **2a**, obtaining the novel mixed complex **4e** ([Cu(phen)_2_(SAL)](ClO_4_)_2_). In contrast with SAL, this complex shows anticancer potency in the sub-micromolar range (82-fold and 1.4-fold higher than SAL and **2a** in A-2780, respectively), induces ER-stress mediated cell death (BiP, and DDIT3 overexpression) and DNA damage in A-2780 and SKOV-3 cells, as evidenced by the massive intracellular production of the phosphorylated histone γ-H2AX. The cytotoxicity of **4e** can be alleviated by co-administration with TUDCA, as previously seen with **2a** and **4a**–**c** [30].

IC_50_ values for the selected compounds are reported in Table 2.

### 2.3. Organic-N Donating Auxiliary Ligands

**Figure 5:**
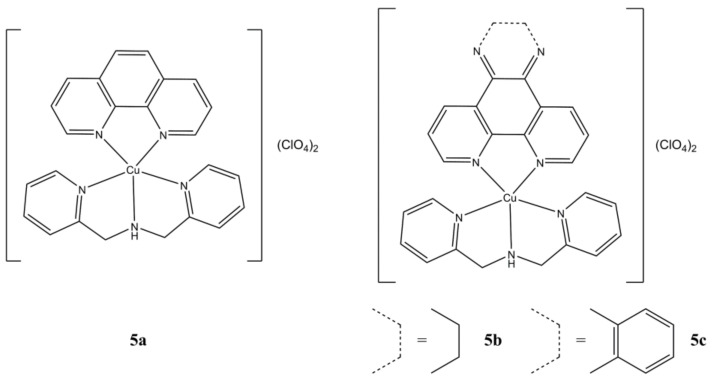
Structures of the [Cu(N-N^2^)(DPA)](ClO_4_)_2_ complexes.

Fantoni et al. have prepared a series of Cu(II)-DPA (DPA is di(2-pycolylamine)) complexes having general formula [Cu(N-N^2^)(DPA)](ClO_4_)_2_ (Figure 5), where N-N^2^ are phen, DPQ and DPPZ [30].

The studied compounds are active against pancreatic (PIN 127, MIA PaCa-2, Panc-1, HPAC) cancer cells at micromolar concentration range. In particular, **5c** shows higher anticancer potency in PIN 127 (5 times) and Panc-1 (3 times) cells compared to the clinical drug oxaliplatin. Ct-DNA binding experiments suggest that these complexes would preferentially interact in the G-C rich regions of the minor groove. Oxidative plasmid DNA damage mediated by OH**^·^** and O_2_^−**·**^ radicals is also observed. The DNA repairing action mediated by endonucleases (Endo III, IV and V) and glycosylases (Fpg) is differently inhibited according to the type of N-N^2^ unit. Interestingly, in association with the repairing hAAG enzyme, the extent of the DNA damage is even increased according to the degree of planarity of the N-N^2^ ligand. IC_50_ values from cell viability studies are reported in Table 3.

### 2.4. Organic-O Donating Auxiliary Ligands

**Figure 6:**
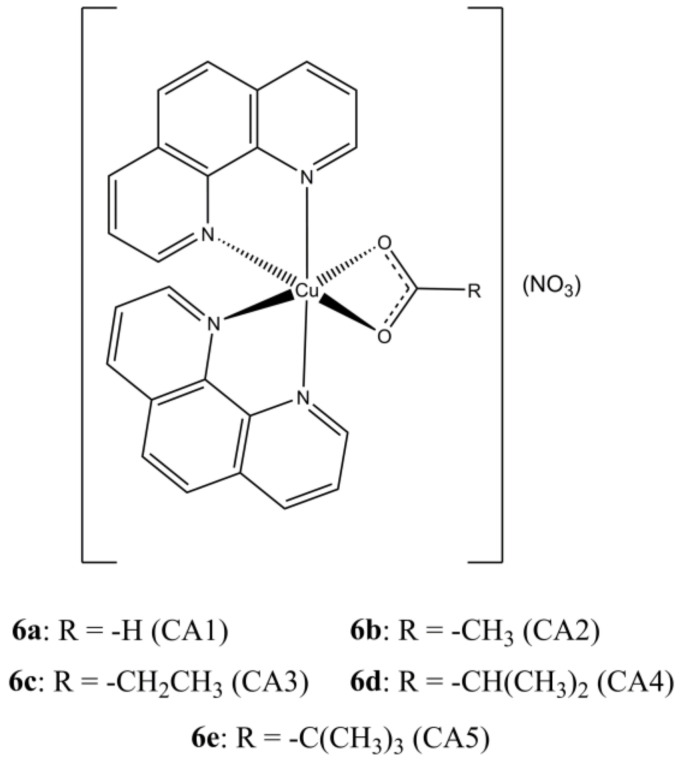
Structures of the [Cu(phen)_2_(CAn)](NO_3_)_2_ complexes.

With the aim of enhancing the target specificity of [Cu(phen)_2_](NO_3_)_2_, Prisecaru et al. introduced some simple carboxylic acids in the [Cu(phen)_2_]^2+^ core obtaining a small library of mixed Cu(II) complexes having general formula [Cu(phen)_2_(CAn)](NO_3_) (Figure 6) [31].

The introduction of an auxiliary carboxylate moiety allowed to increase the affinity towards ct-DNA while reducing that towards albumin. The studied complexes show DNA cleavage activity upon activation by an exogenous reductant (sodium ascorbate) and oxidant (H_2_O_2_), which is believed to arise by a combination of a SOD mimetic activity with OH**^·^** production by Fenton reaction. DNA damage was also evidenced in SKOV-3 cells by accumulation of phosphorylated γ-H2AX. The studied compounds show IC_50_ values in the sub-micromolar range.

**Figure 7:**
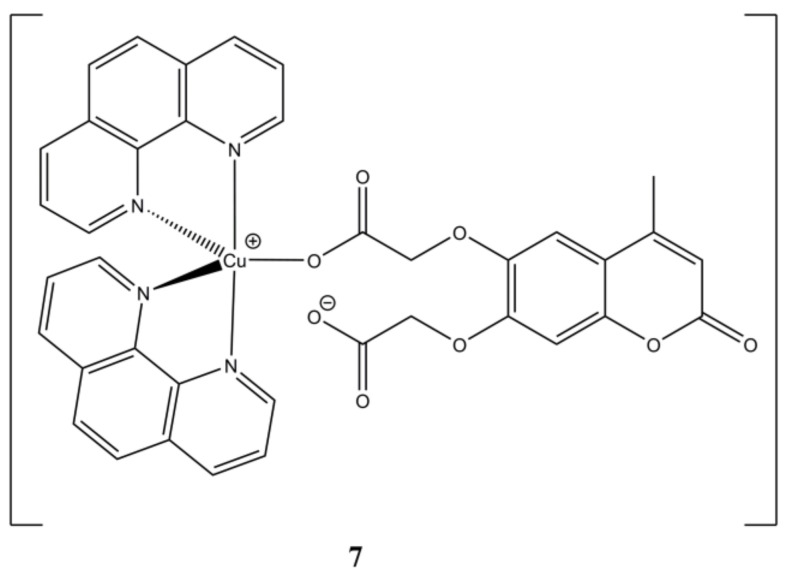
Structures of the [Cu(phen)_2_(4-Mecdoa)] complex.

The mixed Cu(II) complex [Cu(phen)_2_(4-Mecdoa)] (Figure 7) is active towards kidney (A-498) and liver (Hep-G2) cancer cells compared to cisplatin, while being less sensitive towards non-cancerous CHANG cells [32]. However, the same behaviour was not observed on healthy HK-2 lines, this complex appears to interfere with DNA synthesis without any direct interaction with DNA itself. Cell death induction is switchable from apoptosis to necrosis in a dose-dependent manner.

**Figure 8:**
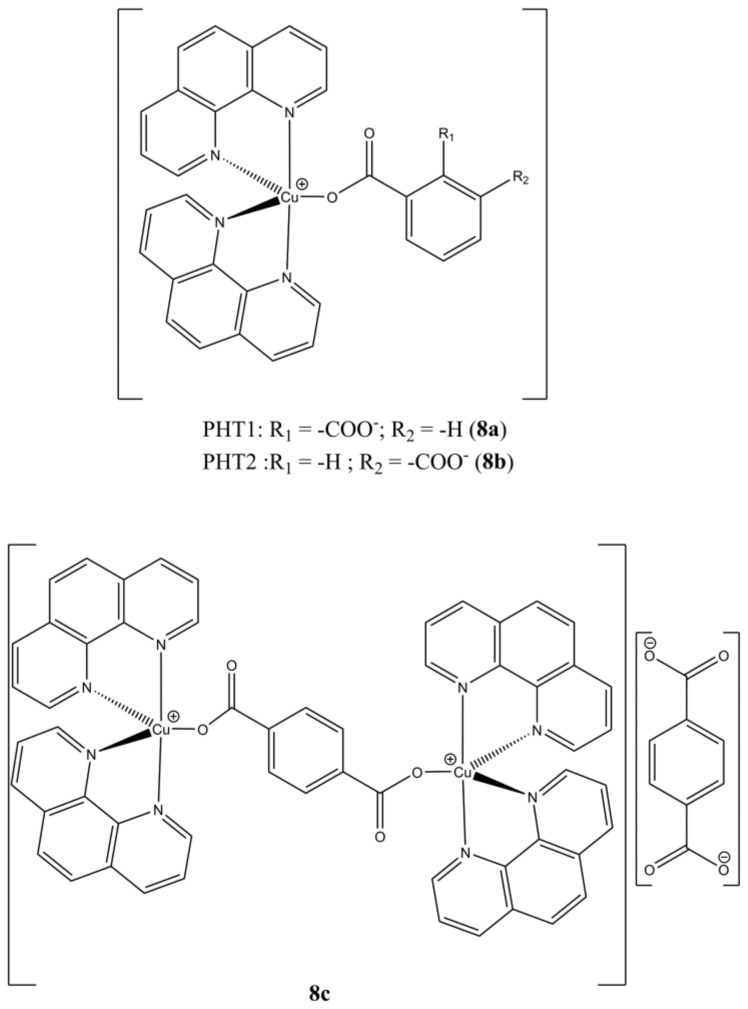
Structures of [Cu(phen)_2_(PHTn)] (**a**,**b**) and [Cu_2_(phen)_4_(µ-PHT3)](PHT3) (**c**) complexes.

Kellet et al. used ftalic acids to prepare the mixed mononuclear [Cu(phen)_2_(PHTn)] (n = 1,2; Figure 8a,b) and binuclear [Cu_2_(phen)_4_(µ-PHT3)](PHT3) (Figure 8c) complexes [33].

These compounds show higher anticancer potencies (at micromolar level) in breast (MCF-7), prostate (DU145) and colon (HT29) cancer cells compared to the approved drugs cisplatin and mitoxantrone, while the Cu(II) complexes of the same PHTn ligands are devoid of any activity. In addition, they can cleave DNA without the presence of exogenous oxidant or reducing agents.

**Figure 9:**
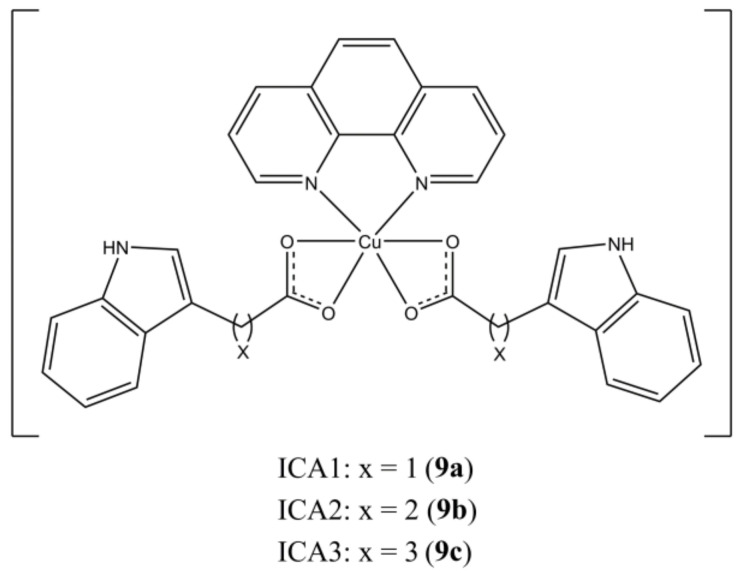
Structures of the [Cu(ICAn)_2_(phen)] complexes.

Zhang et al. reported the synthesis and anticancer activity of mixed Cu(II) complexes having general formula [Cu(ICAn)_2_(phen)], where ICAn are indole 3-carboxylic acids having different alkyl spacers (Figure 9) [34].

These complexes can induce apoptosis in MBA-MDB-231 breast tumours by targeting the Ubiquitin Proteasome Pathway (UPP). It is known that cancer cells are more sensible to UPP inhibition than healthy ones, due to the involvement of this pathway in many carcinogenic processes (e.g., proliferation, apoptosis, and metastasis). In particular, these complexes act at proteasome level by inhibiting the chymotrypsin-like (CT) activity of human 20S proteasome.

**Figure 10:**
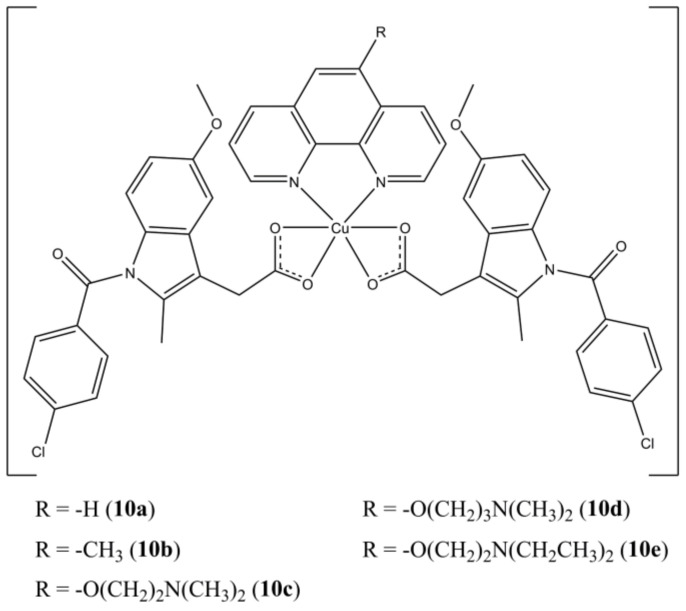
Structures of the [Cu(indomethacin)_2_(5-Rphen)] complexes.

Boodram et al. prepared a panel of mixed complexes [Cu(indomethacin)_2_(5-Rphen)], where the Cu(II) centre is coordinated by the NSAID drug Indomethacin and different 5-substituted phenanthrolines (Figure 10) [35].

The studied complexes are active towards HMLER breast cancer cells at micromolar level. Notably, complexes **10c,d** show selectivity for HMLER-shEcad cells, which possess a higher cancer stem cells (CSCs) content (around 90%) over HLMLER ones (5–8%). The ability of targeting breast CSCs is quite important since these sub-populations of cancer cells are often resistant towards conventional clinical treatments (surgical removal followed by chemo or radiotherapy) and are involved in the resurgence of secondary metastatic breast tumours. The studied compounds can induce DNA damage, as shown by self-activating DNA cleavage activity, cellular ROS production (mainly hydroxyl radicals), and accumulation of γ-H2AX. In addition, they are to target cyclooxygenases by selectively inhibiting COX-2, whose expression is enhanced in several mammary carcinomas.

**Figure 11:**
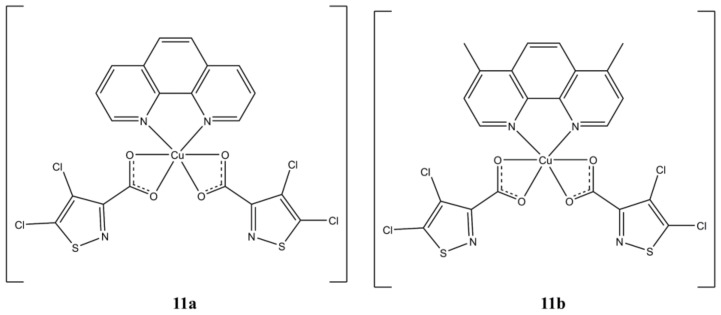
Structures of [Cu(phen)(4,5-dCl-ICA)_2_] (**a**) and [Cu(4,7-diMephen)(4,5-dCl-ICA)_2_] (**b**).

In a series of homo and heteroleptic Cu(II) complexes with 4,5-dichloro-isothiazole 3-carboxylic acid (4,5-dCl-ICA), compounds [Cu(phen)(4,5-dCl-ICA)_2_] and [Cu(4,7-diMephen)(4,5-dCl-ICA)_2_] (Figure 11) proved to be the most potent towards Hep-2 (IC_50_ of 3.06 and 0.97 µM, respectively) and MCF-7 (IC_50_ of 4.2 and 1.8 µM, respectively) cells [36].

The morphological changes observed in cells after treatment with these compounds are indicative of an apoptotic cell death. These complexes can interact with DNA (with K_b_ in agreement with cytotoxic data) but are also able to inhibit the activity of different Cytochrome P450 families in a dose-dependent manner.

**Figure 12:**
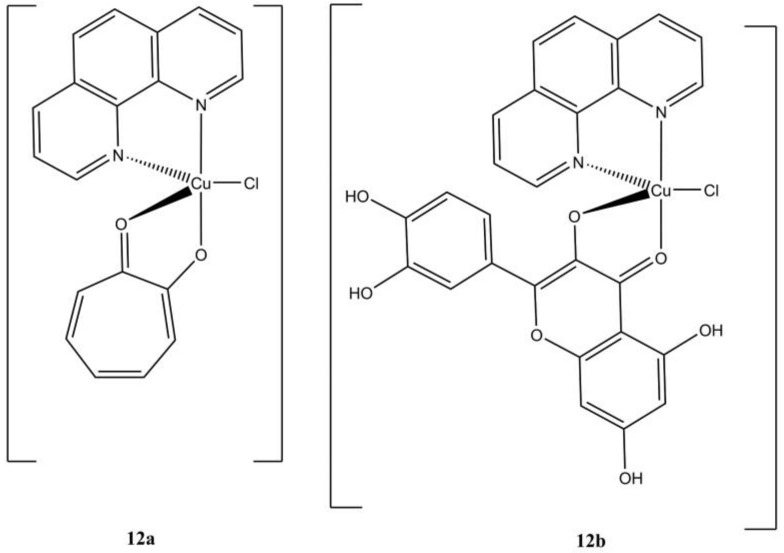
Structures of complexes [Cu(phen)(trop)(Cl)] (**a**) and [Cu(phen)(H_4_Que)(Cl)] (**b**).

The complex [Cu(phen)(trop)(Cl)], bearing a tropolone molecule as auxiliary ligand (Figure 12a), showed the highest anticancer potency (at micromolar level) among a series of binary and ternary Cu(II) tropolone-based complexes [37]. This compound induces cell death in gastric (MGC80-3) cancer cells mainly through a caspase-regulated apoptotic pathway with the induction of ROS production, alteration of the mitochondrial potential, overexpression of many apoptotic signallers (e.g., Bax, cytochrome C, Bak, apaf1). The induction of autophagy in the same cell lines is also observed.

In a screening of novel M(II) (Cu(II), Ni(II), Co(II)) quercetin-based anticancer complexes, Gençkal et al. identified the compound [Cu(phen)(H_4_Que)(Cl)] (Figure 12b) as one of the most promising against MCF-7 and MBA-MDB-231 breast cancers. This compound triggers cell death through caspase-mediated apoptosis, with increasing in cellular ROS content and depolarization of the mitochondrial membrane [38].

The IC_50_ for the compounds discussed in this section are summarized in Table 4.

### 2.5. Organic Mixed Donating Auxiliary Ligands

**Figure 13:**
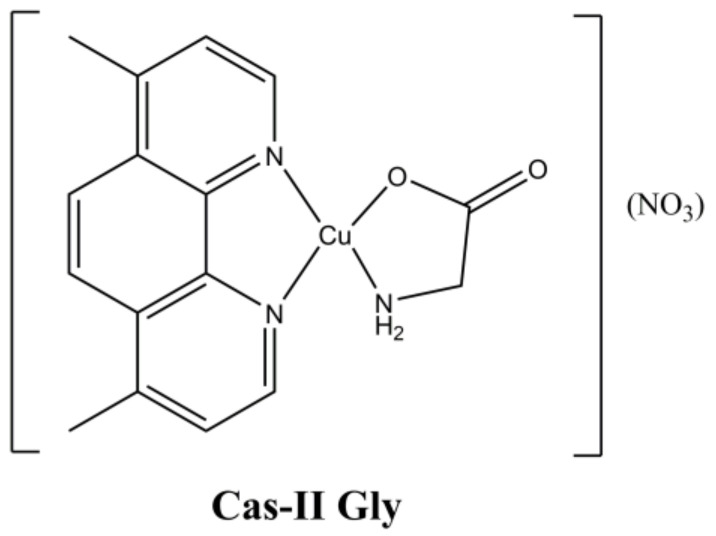
Structure of Cas-II Gly ([Cu(4,7-diMephen)(Gly)](NO_3_)).

The mixed Cu(II)-phenanthroline complex “Cas-II Gly” [Cu(4,7-diMephen)(Gly)](NO_3_) (Figure 13) belongs to the Casiopeinas^®^, a class of complexes having general formula [Cu(N-N)(A-A)](NO_3_) (where “N-N” is a neutral di-imine ligand and “A-A” an amino acid) developed by Ruiz-Azuara et al. [40].

These complexes have been extensively studied on both in vitro and in vivo models, showing that their anticancer activity is exerted through ROS accumulation that causes mitochondrial dysfunction, DNA damage and induces apoptosis [41,42,43,44,45,46,47]. Some of these derivatives have been selected as candidates for Phase I clinical trials [48,49,50].

**Figure 14:**
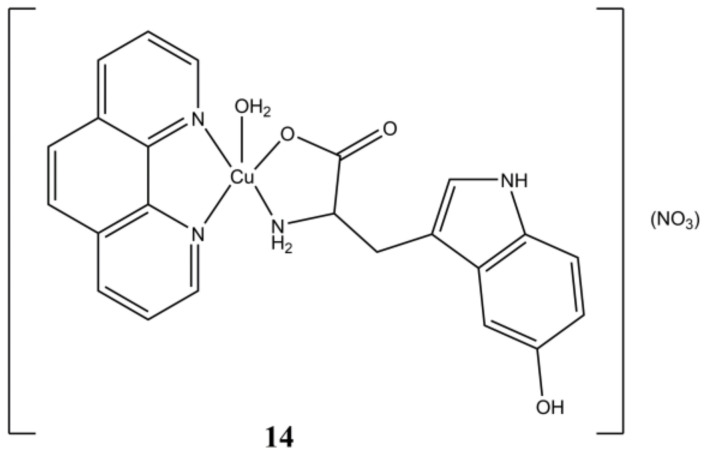
Structure of [Cu(5HTP)(phen)(H_2_O)](NO_3_).

Based on these results, different Cu(II) complexes bearing phen-based ligands and amino acids have been prepared and assayed for their anticancer properties. For instance, the complex [Cu(5HTP)(phen)(H_2_O)](NO_3_) (5HTP is 5-hydroxytryptophan) reported by Naso et al. (Figure 14), shows anticancer activity in the micromolar range (IC_50_ of 3.6 µM) in A-549 cells, while being devoid of any cytotoxic activity in healthy MRC-5 cells.

The authors demonstrated how the anticancer activity of this compound is exerted through cellular ROS production, GSH depletion and alteration of mitochondrial potential. The complex shows also antimetastatic activity on A-549 cells with inhibition of cells adhesion, migration, and invasion [51].

**Figure 15:**
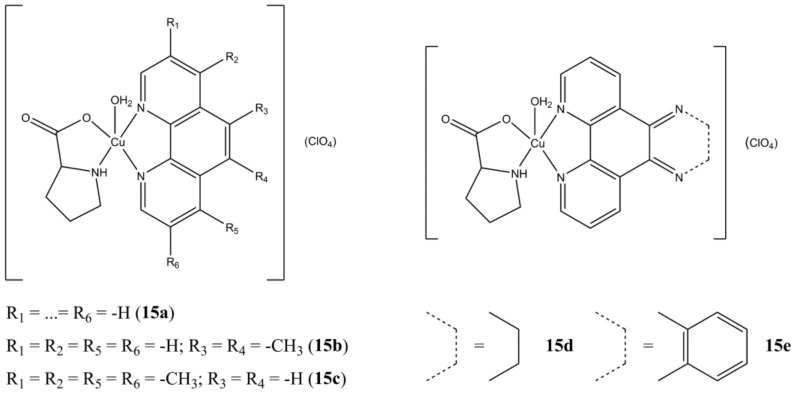
Structures of “phen-based” [Cu(L-pro)(N-N^3^)(H_2_O)_n_](ClO_4_) complexes.

Karpagam et al. prepared a series of Cu(II) Proline-based complexes having general formula [Cu(L-pro)(N-N^3^)(H_2_O)_n_](ClO_4_) (n = 0,1) [52]. The insertion of a phenanthroline-based ligand (Figure 15) generally increases the anticancer potency (A-549 cell lines) compared to the bipyridine-based analogues. Compounds **15b** and **15d** proved to be the most promising in terms of potency (IC_50_ of 1.4 and 1.3 µM, respectively), showing the importance of the substitution pattern in the 5th and 6th position of the phenanthroline backbone. Cell death induction mainly take place through apoptosis induction, with cellular overexpression of ROS.

**Figure 16:**
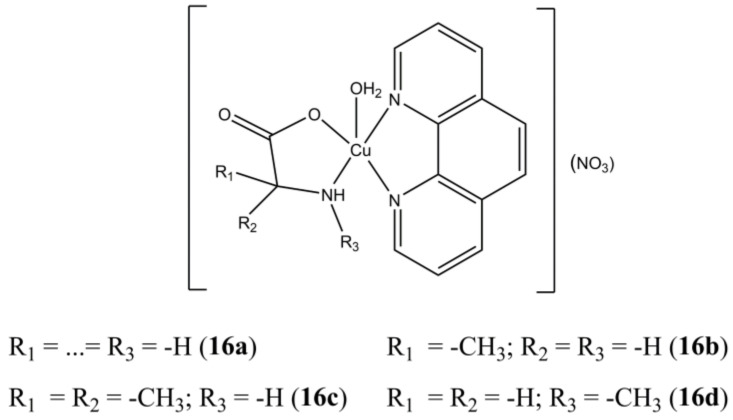
Structures of [Cu(phen)(AAn)(H_2_O)](NO_3_) complexes.

The panel of complexes [Cu(phen)(AAn)(H_2_O)](NO_3_) (“AAn” are glycine and different methylated glycine derivatives) developed by Seng et al. (Figure 16) show selectivity towards cancerous nasopharyngeal HK1 cells rather than healthy NP69 ones [53]. Derivatives **16c** and **16d** are the most active (IC_50_ of 2.2 µM for both) and selective (Selectivity Index SI > 11.4). The studied compounds show moderate affinity towards ct-DNA, with preferential interaction towards G-C sites. Inhibition towards DNA Topo1 in a dose-dependent manner is also evidenced.

**Figure 17:**
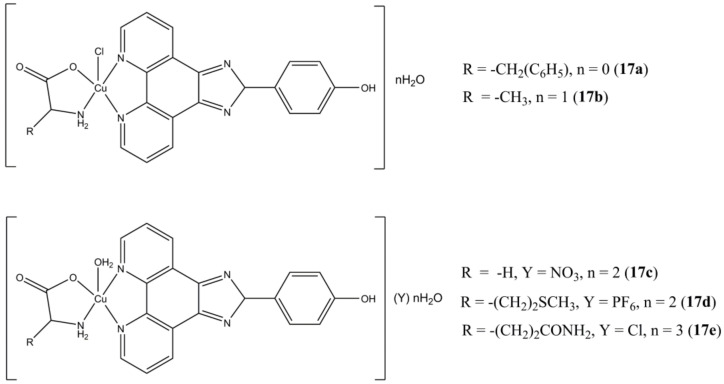
Structures of the Cu(II)–(OH-PIP) based complexes.

Li et al. prepared a series of heteroleptic Cu(II) complexes having different α-amino acids and the fused phenanthroline derivative OH-PIP (4-(2H-imidazo [4,5-f][1,10]phenanthrolin-2-yl)phenol) as ligands (Figure 17) [54].

These compounds show anticancer potency at micromolar/sub-micromolar concentrations against triple negative (MCF-7, MDA-MB-231, CAL-51) breast cancers. The best activity was observed for CAL-51, with IC_50_ values in the 0.082–0.69 μM range. The complexes here reported show higher potencies compared to clinically approved carboplatin and induces apoptotic cell death by interfering at UPP level (inhibition of CT-like activity of 20S proteasome, PARP cleavage). The most promising complexes of the series (**17c** and **17e**) are also able to significantly reduce the percentage of different triple negative stem cancer sub-populations.

**Figure 18:**
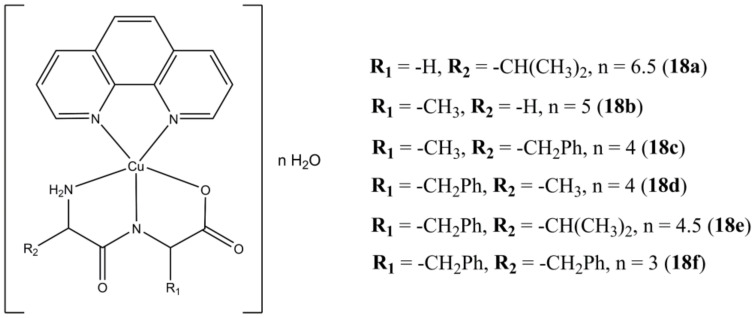
Structure of the [Cu(L-dipeptide)(phen)]·nH_2_O complexes.

Facchin et al. have been focusing on the design and synthesis of ternary Cu(II) complexes bearing phen-based molecules and L-dipeptides [55]. For instance, compounds of general formula [Cu(L-dipeptide)(phen)]·nH_2_O (Figure 18) have been screened for anticancer activity in cervical (Hela), breast (MCF-7) and lung (A-549) cancer cells showing anticancer potencies in the micromolar concentration range [56]. Preliminary studies on Albumin and ct-DNA interaction with the studied molecules show low to moderate affinity towards these biomolecules, with ct-DNA binding constants that appear to not be correlated with neither cytotoxicity nor lipophilicity. Interestingly, the author experimentally evaluated the lipophilicity of these molecules, pointing out how this parameter is influenced not only by the number and types of apolar groups (e.g., methylene, phenyl rings), but also on the spatial arrangement of the complexes, as evidenced for compounds **18c** and **18d**, where the auxiliary ligands are the Ala-Phe and Phe-Ala L-dipeptides, respectively.

**Figure 19:**
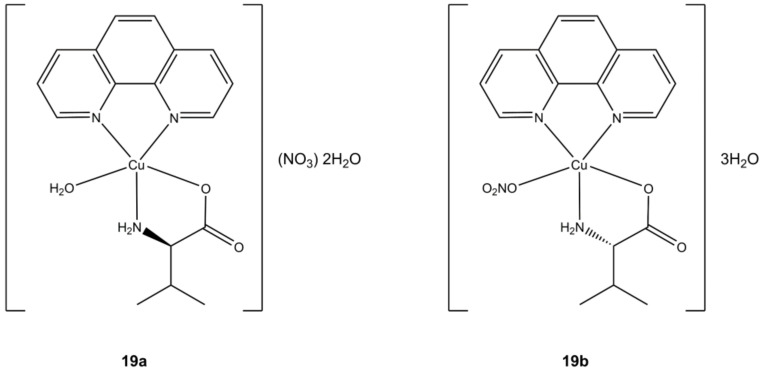
Structure of the [Cu(L-dipeptide)(phen)]·nH_2_O complexes.

The chiral Cu(II) complexes [Cu(phen)(L-Val)(OH_2_)](NO_3_)∙2H_2_O (Figure 19a) and [Cu(phen)(D-Val)(ONO_2_)]∙3H_2_O (Figure 19b) of Arjmand et al. shows anticancer potency in the micromolar order on breast (MCF-7), pancreatic (BxPC3, AsPC1) and liver (Huh7) cancer cells. Notably, these compounds are able to target G-quadruplex DNA (G4 DNA) by cleaving it at selective sites. In general, compounds that can interfere at G4 DNA level (G4 ligands) are preferred since this interaction will result in telomerase inhibition, whose activity is up regulated in cancer cells while being silent in healthy ones. The inhibition of this enzymatic pathway will result in accumulation of shorter telomers and subsequent induction of apoptosis [57].

**Figure 20:**
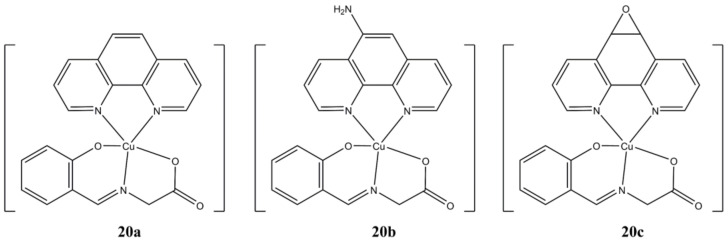
Structures of the [Cu(Sal-Gly)(N-N^4^)] complexes.

Acilan et al. prepared a series of [Cu(Sal-Gly)(N-N^4^)] complexes (Figure 20), where Sal-Gly is the *O,N,O* Schiff Base obtained from Salicylaldehyde and Glycine, while the N-N^4^ ligands are phen, 1,10-phenanthroline-5-amine, 5,6-diepoxy-1,10-phenanthroline [58].

The complexes show dose and time-dependent micromolar cytotoxicity towards cancerous (A-549, HCT-116, HeLa, MBA-MB-231 and SHSY5Y) cells, while generally being less active against healthy HASMC1 and HASMC2 cells. Compound **20b** appears to be the most promising in terms of both anticancer potency and selectivity. Rapid apoptotic cell death in HCT-116 and HeLa cells was detected with induction of ROS production, depolarization of mitochondrial membrane, depletion of GSH and cellular DNA damage (γ-H2AX expression). Interestingly, gene-knockdown experiments on HeLa cells have shown that apoptotic cell death doesn’t rely on p53 status, thus supporting the hypothesis that these complexes might be potent also in p53 deficient cancer cell lines.

**Figure 21:**
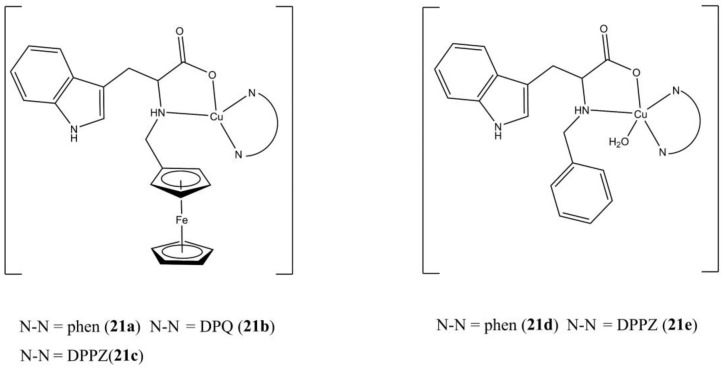
Structures of the phenanthroline-based [Cu(Fc-Trp)(N-N^5^)](ClO_4_) (**a**–**c**) and [Cu(Ph-Trp)(N-N^5^)(OH_2_)](ClO_4_) (**d**,**e**) complexes.

Goswami et al. has prepared and studied the photo-toxicity of a series of mixed Cu(II) complexes having neutral N-N^5^ ligands (bipy, phen, DPQ, DPPZ), and tryptophan-based (Fc-Trp and Ph-Trp) ligands (Figure 21) [59].

These complexes generally possess enhanced anticancer potencies when exposed to visible light in both He-La (e.g., compound **21c** IC_50_ of 8.95 and 1.29 µM in the dark and under visible light, respectively) and MCF-7 cells (e.g., compound **21c** IC_50_ of 2.99 and 0.65 µM in the dark and under visible light, respectively). Cell death induction takes place through caspase-independent apoptotic mechanism, with higher apoptotic percentage when cells are exposed under visible light. The negligible anticancer activity observed for the [Zn(Fc-Trp)(DPPZ)](ClO_4_) complex in HeLa cells (IC_50_ > 80 µM) suggest the importance of the Cu(II) centre in exerting the anticancer activity. The studied compounds interact with ct-DNA as groove binders and can induce DNA cleavage through OH**^·^** production.

**Figure 22:**
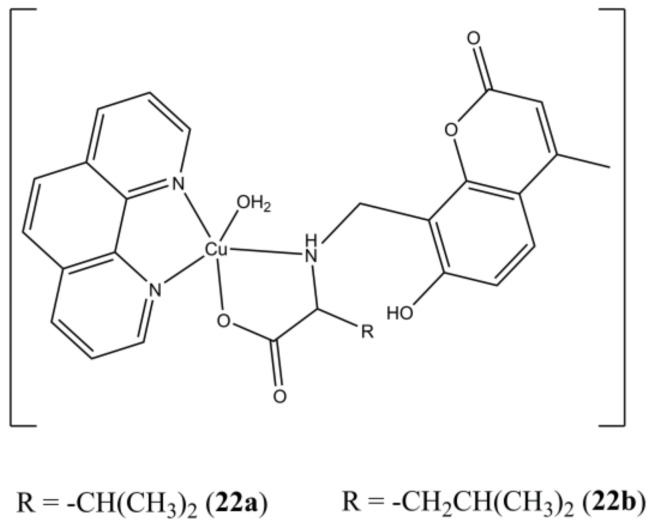
Structures of the [Cu(MCVH)(phen)(OH_2_)](ClO_4_) (**a**) and [Cu(MCLH)(phen)(OH_2_)](ClO_4_) (**b**) complexes.

The two [Cu(HMCX)(phen)(OH_2_)](ClO_4_) complexes (Figure 22), where the two H_2_MCX ligands are obtained by coupling L-Valine (X = V) and L-Leucine (X = L) with 7-hydroxy-4-methylcoumarin, intercalates ct-DNA and possess micromolar anticancer activity against human prostate (PC3), liver (L02) and myeloid leukemia (HL-60) cancer cells [60].

**Figure 23:**
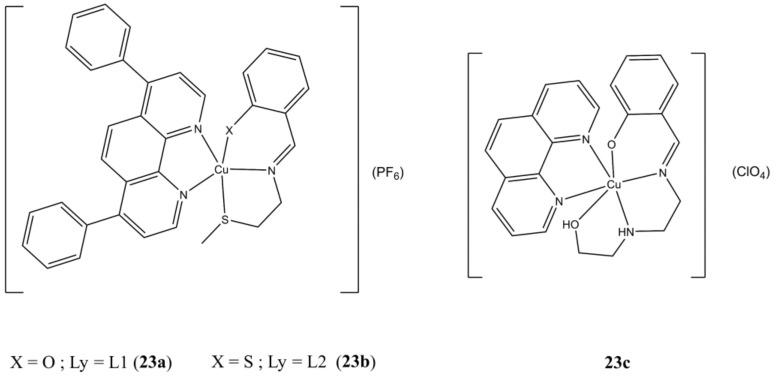
Structures of complexes [Cu(Ly)(bathophen)](PF_6_) (**a**,**b**) and [Cu(tdp)(phen)](ClO_4_) (**c**).

The two [Cu(Ly)(bathophen)](PF_6_) (Figure 23a,b) complexes (Ly are *O/S,N,S* Schiff bases, batophen is 4,7-diphenil-1,10-phenanthroline) show sub-micromolar anticancer potencies (IC_50_ in the 0.21–0.32 µM range) against both HMLER and HMLER-shEcad cells, thus proving to be active against breast CSC subpopulations too. Both complexes show higher anticancer potencies compared to approved drugs cisplatin and salinomycin. Moreover, they can reduce the number and dimensions of 3D HMLER-shEcad mammospheres [61].

The Cu(II) complex [Cu(tdp)(phen)](ClO_4_) (Figure 23c), where Htdp is 2-[(2-(2-hydroxyethylamino)-ethylimino)methyl]phenol, shows IC_50_ in the micromolar range in both MCF-7 and MDA-MB-231 cancer cells. This compound induces alteration of mitochondrial potential, ROS overexpression, cell-cycle arrest (at S- and G2/M phases) and cellular DNA damage followed by apoptosis, which can turn to necrosis at higher concentrations or longer durations of treatments. Interestingly, the Bax/Bcl-2 expression ratios were differently affected in MCF (p53^+^, ER^+^) and MDA-MB-231 (p53^−^, ER^−^), thus suggesting a potential genotype-selective mechanism mediated by the p53 protein, which still need to be clarified though [62].

**Figure 24:**
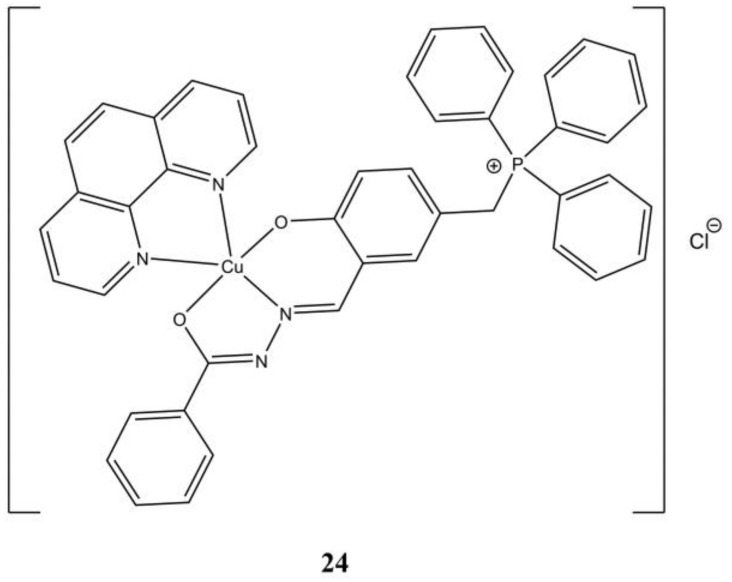
Structure of the [Cu(phen)(L3)]Cl complex.

In a series of Cu(II) complexes having 5-(triphenylphosphoniummethyl)-salicylaldehyde benzoylhydrazone] chloride (L3) and *N*,*N*-diimine ligands, [Cu(phen)(L3)]Cl (Figure 24) proved to be the most promising against A-549 (IC_50_ of 4.2 µM) and PC-3 (IC_50_ of 3.2 µM) cancer cells. The studied complexes are able to interfere at DNA level through inhibition of DNA Topoisomerase-I (Topo-I) [63].

**Figure 25:**
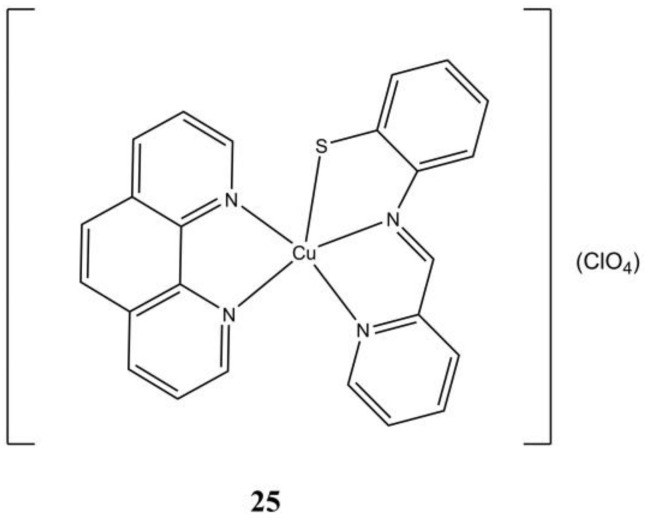
Structure of the [Cu(pabt)(phen)](ClO_4_) complex.

The complex [Cu(pabt)(phen)](ClO_4_) (Figure 25), where Hpabt is *N*-(2-mercaptophenyl)-2′-pyridylmethylenimine, shows anticancer activity at micromolar level against A-549 and A-431(IC_50_ of 5.26 and 5.41 µM, respectively) cancer cells, while being less cytotoxic against healthy L132 and HaCaT cells (IC_50_ of 7.47 and >10 µM, respectively). This compound shows intercalates ct-DNA showing good binding affinity and triggers cell death in A-549 through apoptosis [64].

IC_50_ values for the compounds here reported are shown in Table 5.

### 2.6. Polynuclear Complexes

Prisecaru et al. focused their attention on the complex **8c** previously synthesized showing that its ability of cleaving DNA takes place oxidatively and in a non-specific manner. The complex shows comparable potency on both Platinum-resistant SKOV-3 (IC_50_ of 6.7 µM) cells and healthy HS-832 ones (IC_50_ of 4.5 µM) but is also 8 times more active than clinical drug Mitoxantrone on SKOV-3 cells (IC_50_ of 54.5 µM). Cellular ROS production in A-549 cells up to nanomolar concentration level was also observed [39].

**Figure 26:**
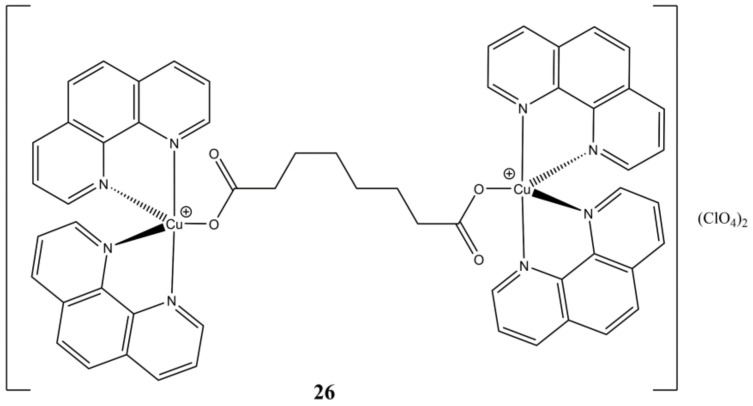
Structure of the [Cu_2_(μ-oda)(phen)_4_](ClO_4_)_2_ complex.

The same authors have evaluated the anticancer properties of [Cu_2_(μ-oda)(phen)_4_](ClO_4_)_2_ (Figure 26), where the bridging ligand “oda” is octanedioic acid.

This complex shows time and concentration dependent (from low micromolar to nanomolar at 24 and 96 h of treatment, respectively) cytotoxic activity towards colorectal HT29, SW480 and SW620 cancer cells. In vivo drug tolerability studies on *G. Melonella* larvae shows that the complex is better tolerated than cisplatin in the 200–1000 µg/mL concentration range. The reported compound shows good ct-DNA affinity, self-activating DNA cleavage ability in presence of oxygen, along with artificial SOD activity and cellular ROS induction ability. The authors combined these experimental evidence and proposed a model of DNA cleavage based on the formation of reduced [Cu(phen)_2_]^+^ complex and a π carboxylate radical, which can oxidatively damage DNA in multiple ways. For instance, the carboxylate radical and/or its alkyl product of decarboxylation might abstract a hydrogen from the DNA phosphodiester backbone, while the [Cu(phen)_2_]^+^ complex might induce DNA breaks through aerobic-generated oxo and hydroxo complexes [65].

**Figure 27:**
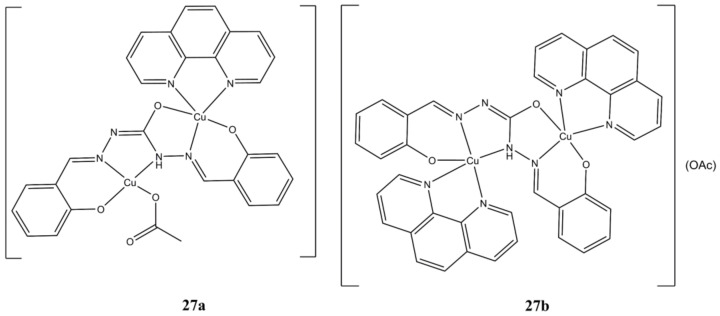
Structure of complexes [Cu(phen)(SCH)Cu(OAc)] (**a**) and [Cu_2_(SCH)(phen)_2_](OAc) (**b**).

Parsekar et al. obtained the two binuclear Cu(II) complexes [Cu(phen)(SCH)Cu(OAc)] and [Cu_2_(SCH)(phen)_2_](OAc) by mixing Cu(OAC)_2_, phen and the SCH ligand (1,5-bis(salicylidene)carbohydrazide) in different molar ratios (Figure 27) [66].

Both complexes are cytotoxic (micromolar level) towards cancerous A-549 and MCF-7 cancer cells and to a lower extent towards healthy HaCaT cells. Cellular growth is arrested mainly at G2/M phase and induction of cell death can switch from early to late apoptosis/necrosis in a dose-dependent manner. Both complexes can intercalate ct-DNA and inducing both hydrolytic and oxidative DNA cleavages. Interestingly, increase in ROS production is evidenced in A-549 while the opposite is observed in MCF-7 lines.

**Figure 28:**
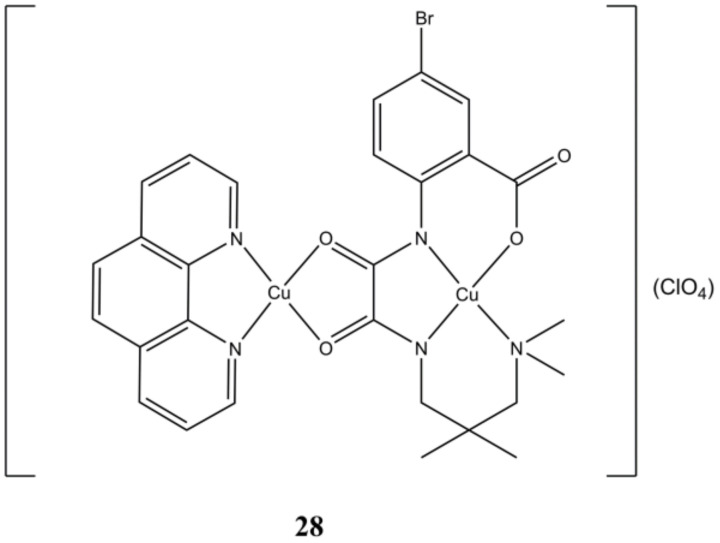
Structure of the [Cu_2_(L4)(phen)](ClO_4_) complex.

The binuclear [Cu_2_(L4)(phen)](ClO_4_) complex (Figure 28) can be reduced by GSH, and in turn catalyze the conversion of H_2_O_2_ to hydroxy radicals, as demonstrated at both spectroscopic and at cellular level. This complex is cytotoxic at micromolar level towards 4T1, A-549, HepG2 and MCF-7 cancer cells and to a lower extent towards healthy COS-7 cells. This compound arrest cell cycle at G0/G1 phase and induces cell death through apoptosis. The dual ability of **26** in depleting the cellular antioxidant GSH and producing ROS through H_2_O_2_ (often overexpressed in tumorigenic cells) makes this compound a potential candidate for applications in chemo-dynamic therapy [67].

IC_50_ values for the polynuclear complexes here reported are summarized in Table 6.

## 3. Conclusions

**Figure 29:**
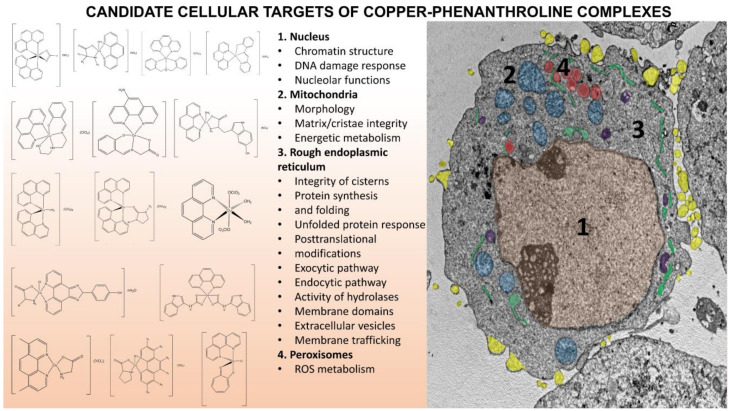
The candidate cellular structures and molecular mechanisms interfering with copper-phen complexes visualized in the A-2780 cancer cell.

Mixed Cu(II) complexes bearing phenanthroline-based ligands have potential applications in anticancer chemotherapy, as evidenced by the numerous examples reported in this review. Figure 29 summarizes the principal action mechanisms observed for the copper phen-based complexes so far shown. As can be seen, similar molecules can exert very different biological actions, just as structurally different molecules can act according to the same mechanism of action. In many cases, a molecule can exert its cytotoxic action through multiple mechanisms; the predominance of one or more of them can depend on multiple factors, not always predictable. Comparisons between the different molecules can provide new druggable cellular targets or contribute to understanding of molecular mechanisms in cell.

The different coordination numbers and geometries adopted by Cu(II) with many classes of ligands has certainly the advantage of extending the number of novel chemical entities that might be screened for their biological activity but also makes more difficult to draw generalized Structure Activity Relationships (SAR). However, some considerations could be made: (i) Cu(II) complexes are generally more potent by the related Cu(II) salts or ligands, thus proving the importance of the complex itself in exerting the anticancer activity; (ii) the Cu(II) centre is fundamental for exerting the desired biochemical properties, due to its involvement in ROS production; (iii) the planarity of the N-N moiety generally increases the anticancer potency (e.g., complexes having phen ligands are more potent than the bipy-based ones).

As regards the auxiliary ligands, their choice is commonly made under two different perspectives, such as their known biochemical properties (e.g., NSAIDs, natural compounds, cytotoxic molecules) or their ability in targeting DNA. However, results achieved from biological assays are not always straightforward, since the activity of the ligand alone could be significantly altered when involved in a metal complex (e.g., Salubrinal is cytoprotective towards ER-stress, while the **4e** complex is cytotoxic).

Rational design of both complexes and biochemical experiments are often performed taking the DNA as the primary (and only) molecular target. As clearly demonstrated in various examples, the way these compounds exert their biochemical properties is not restricted to the binding and/or cleavage of DNA. For this reason, more efforts should be made in evaluating the interactions with other potential biological targets and pathways, in order to better clarify the molecular mechanisms adopted.

Despite the promising in vitro anticancer properties reported there are a few in vivo studies that can confirm the biological activity observed in cellular models and evaluate the ADMET (Absorption, Distribution, Metabolism, Excretion, Toxicity) properties of the studied compounds. More efforts in this direction should be made to evaluate the possible lead compounds that can be further evaluated for pre-clinical studies.

In summary, mixed Cu(II) complexes bearing phenanthroline-based ligands are promising compounds that can be targeted to various molecular pathways and mechanisms in cancer cells.

## Figures and Tables

**Table 1:** IC_50_ values of compounds **2a**–**3d**.

Compound	Cell Line (IC_50_, µM)	Reference
**2a**	24 h: CCRF-CEM (1.25 ± 0.2), A-2780 (0.94 ± 0.04), CCRF-SB (0.50 ± 0.5), SKMES-1 (0.93 ± 0.6), DU-145 (1.6 ± 0.2), CRL-7065 (2.20 ± 0.03), HEK-293 (1.098), HEP-G2 (1.05 ± 0.01), A-2780 (0.5916)	[24,27,29]
**2b**	24 h: CCRF-CEM (3.2 ± 0.1), CCRF-SB (1.4 ± 0.1), SKMES-1 (1.9 ± 0.1), DU-145 (2.6 ± 0.3), CRL-7065 (1.34 ± 0.03), HEP-G2 (2.90 ± 0.03),	[24,25]
**2c**	24 h: CCRF-CEM (0.80 ± 0.02), CCRF-SB (0.42 ± 0.01), SKMES-1 (1.20 ± 0.01), DU-145 (1.16 ± 0.02), HEP-G2 (0.67 ± 0.02), CRL-7065 (2.47 ± 0.02)	[25]
**2d**	24 h: CCRF-CEM (3.80 ± 0.02), CCRF-SB (1.21 ± 0.01), SKMES-1 (3.10 ± 0.01), DU-145 (4.10 ± 0.01), HEP-G2 (1.70 ± 0.02), CRL-7065 (6.30 ± 0.01)	[25]
**2e**	24 h: CCRF-CEM (0.09 ± 0.04), CCRF-SB (0.07 ± 0.03), SKMES-1 (0.54 ± 0.01), DU-145 (0.93 ± 0.01), HEP-G2 (1.20 ± 0.01), CRL-7065 (1.34 ± 0.03)	[25]
**2f**	24 h: CCRF-CEM (0.18 ± 0.01), CCRF-SB (0.20 ± 0.01), SKMES-1 (2.25 ± 0.02), DU-145 (4.00 ± 0.02), HEP-G2 (3.60 ± 0.01), CRL-7065 (3.10 ± 0.01)	[25]
**3a**	48 h: HeLa (45.37 ± 1.98), SKOV-3 (32.28 ± 2.43), HK-2 (65.24 ± 2.68)	[28]
**3b**	48 h: HeLa (33.60 ± 1.53), SKOV-3 (21.81 ± 1.57), HK-2 (57.25 ± 1.43)	[28]
**3c**	48 h: HeLa (15.97 ± 1.67), SKOV-3 (12.45 ± 1.36), HK-2 (36.18 ± 1.82)	[28]
**3d**	48 h: HeLa (7.32 ± 1.35), SKOV-3 (6.56 ± 1.28), HK-2 (21.57 ± 1.26)	[28]

**Table 2:** IC_50_ values of compounds **4a**–**4e**.

Compound	Cell line (IC_50_, µM)	Reference
**4a**	24 h: CCRF-CEM (0.8 ± 0.8), CCRF-SB (0.70 ± 0.7), SKMES-1 (0.85 ± 0.04), DU-145 (1.6 ± 0.2), HEK-293 (1.181), A-2780 (0.4342), SKOV-3 (1.524)	[24,27]
**4b**	24 h: CCRF-CEM (0.8 ± 0.8), CCRF-SB (0.60 ± 0.5), SKMES-1 (0.97 ± 0.7), DU-145 (3.6 ± 0.4), HEK-293 (1.293), A-2780 (0.4385), SKOV-3 (1.381)	[24,27]
**4c**	24 h: CCRF-CEM (1.1 ± 0.1), CCRF-SB (1.3 ± 0.1), SKMES-1 (0.9 ± 0.1), DU-145 (1.5 ± 0.2), HEK-293 (1.097), A-2780 (0.2865), SKOV-3 (1.524)	[24,27]
**4d**	24 h: CCRF-CEM (0.90 ± 0.09), CCRF-SB (0.80 ± 0.07), SKMES-1 (0.7 ± 0.1), DU-145 (1.50 ± 0.05)	[24]
**4e**	24 h: A-2780 (0.68 ± 0.05)	[29]

**Table 3:** IC_50_ values of compounds **5a**–**5c**.

Compound	Cell Line (IC_50_, µM)	Reference
**5a**	120 h: PIN127 (1.05 ± 0.15), MIA PaCa-2 (2.97 ± 1.27), Panc-1 (1.40 ± 0.33), HPAC (1.27 ± 0.56)	[30]
**5b**	120 h: PIN127 (0.93 ± 0.02), MIA PaCa-2 (1.06 ± 0.47), Panc-1 (0.57 ± 0.19), HPAC (0.44 ± 0.05)	[30]
**5c**	120 h: PIN127 (0.20 ± 0.01), Panc-1 (0.48 ± 0.13), HPAC (0.32 ± 0.12)	[30]

**Table 4:** IC_50_ values of compounds **6a**–**12b**.

Compound	Cell Line (IC_50_, µM)	Reference
**6a**	24 h: PC3 (>200), SKOV-3 (2.28 ± 0.14), PNT1A (52.01 ± 7.02). 96 h: PC3 (4.32 ± 1.14), SKOV-3 (1.41 ± 0.07), PNT1A (1.98 ± 0.23)	[31]
**6b**	24 h: PC3 (>200), SKOV-3 (3.04 ± 0.19), PNT1A (75.02 ± 30.29). 96 h: PC3 (5.41 ± 3.01), SKOV-3 (2.05 ± 0.14), PNT1A (1.51 ± 0.18)	[31]
**6c**	24 h: PC3 (26.09 ± 1.56), SKOV-3 (3.02 ± 0.10), PNT1A (27.60 ± 11.37). 96 h: PC3 (2.25 ± 0.22), SKOV-3 (2.07 ± 0.19). 96 h, PNT1A (2.62± 0.66)	[31]
**6d**	24 h: PC3 (52.00 ± 12.70), SKOV-3 (2.86 ± 1.36), PNT1A (23.89 ± 3.74). 96 h: PC3 (2.31 ± 0.08), SKOV-3 (1.91 ± 0.09), PNT1A (2.05 ± 0.07)	[31]
**6e**	24 h: PC3 (3.26 ± 4.34), SKOV-3 (3.59 ± 0.10), PNT1A (14.56 ± 2.44). 96 h: PC3 (3.96 ± 1.45), SKOV-3 (1.78 ± 0.05), PNT1A (1.90 ± 0.21)	[31]
**7**	96 h: CHANG (11 ± 1.8), A-498 (2.0 ± 1.2), HK-2 (2.8 ± 0.8), HEP-G2 (1.3 ± 0.8)	[32]
**8a**	24 h: MCF-7 (44.9 ± 7.0), DU-145 (11.6 ± 4.5), HT29 (6.0 ± 0.4)	[33]
**8b**	24 h: MCF-7 (41.2 ± 1.4), DU-145 (10.6 ± 2.2), HT29 (5.8 ± 0.2)	[33]
**8c**	24 h: MCF-7 (7.9 ± 0.4), DU-145 (5.7 ± 0.2), HT29 (5.4 ± 0.3), SKOV-3 (6.7 ± 0.4), HS-832 (4.5 ± 0.2)	[33,39]
**9a**	24 h: MDA-MB-231 (4.20), MCF-7 (5.21)	[34]
**9b**	24 h: MDA-MB-231 (4.71), MCF-7 (6.29)	[34]
**9c**	24 h: MDA-MB-231 (5.31), MCF-7 (6.82)	[34]
**10a**	72 h: HMLER (4.4 ± 0.1), HMLER-shEcad (4.3 ± 0.1), 96 h: mammosphere (n.d.)	[35]
**10b**	72 h: HMLER (2.5 ± 0.2), HMLER-shEcad (2.5 ± 0.1), 96 h: mammosphere (n.d.)	[35]
**10c**	72 h: HMLER (7.5 ± 1.4), HMLER-shEcad (2.7 ± 0.2), 96 h: mammosphere (16.6 ± 0.6)	[35]
**10d**	72 h: HMLER (7.4 ± 0.3), HMLER-shEcad (2.2 ± 0.5), 96 h: mammosphere (13.8 ± 0.8)	[35]
**10e**	72 h: HMLER (6.9 ± 1.4), HMLER-shEcad (4.2 ± 0.6), 96 h: mammosphere (26.3 ± 1.3)	[35]
**11a**	48 h: Hep2 (3.06 ± 0.07), MC7 (4.2 ± 0.2)	[36]
**11b**	48 h: Hep2 (0.97 ± 0.03), MC7 (1.8 ± 0.3)	[36]
**12a**	48 h: A-549 (4.5 + 0.1), Bel-7402 (4.5 ± 0.4), MGC80-3 (3.5 ± 0.9), T24 (4.2 ± 0.1), SKOV-3 (5.3 ± 0.6), NCI-H460 (4.3 ± 1.0), HL-7702 (4.8 ± 0.6)	[37]
**12b**	24 h: MCF-7 (20.0), MDA-MB-231 (10.2). 48 h: MCF-7 (2.4), MDA-MB-231 (5.4)	[38]

**Table 5:** IC_50_ values of compounds **Cas-II Gly–25**.

Compound	Cell Line (IC_50_, µM)	Reference
**Cas-II Gly**	24 h: Hela (1.33), HCT-5 (3.7), SKL-U (4.97), MDA-MB-231 (1.55), SK-N-SH (18), MCF-7 (2.1), HCT-15 (2), HeLa (5.5), SiHa (5.5), CHP-212 (31.5), Lymphocytes (1720)	[46,47]
**14**	24 h: A-549 (3.6), MRC-5 (>100)	[51]
**15a**	24 h: A-549 (10 ± 0.01)	[52]
**15b**	24 h: A-549 (1.4 ± 0.20)	[52]
**15c**	24 h: A-549 (15.5 ± 0.05)	[52]
**15d**	24 h: A-549 (1.3 ± 0.05)	[52]
**15e**	24 h: A-549 (9.7 ± 0.20)	[52]
**16a**	24 h: HK1 (5.2), NP-69 (>25)	[53]
**16b**	24 h: HK1 (3.9), NP-69 (13.8)	[53]
**16c**	24 h: HK1 (2.2), NP-69 (>25)	[53]
**16d**	24 h: HK1 (2.2), NP-69 (>25)	[53]
**17a**	24 h: CAL-51 (0.52 ± 0.02), MDA-MB-231 (18.89 ± 1.23), MCF-7 (30.88 ± 2.56)	[54]
**17b**	24 h: CAL-51 (0.080 ± 0.004), MDA-MB-231 (8.35 ± 0.55), MCF-7 (17.08 ± 2.64)	[54]
**17c**	24 h: CAL-51 (0.37 ± 0.04), MDA-MB-231 (10.98 ± 0.95), MCF-7 (25.59 ± 2.10)	[54]
**17d**	24 h: CAL-51 (0.69 ± 0.04), MDA-MB-231 (4.92 ± 0.36), MCF-7 (18.99 ± 1.54)	[54]
**17e**	24 h: CAL-51 (0.27 ± 0.02), MDA-MB-231 (9.33 ± 0.84), MCF-7 (20.32 ± 2.01)	[54]
**18a**	48 h: HeLa (15), MCF-7(18), A-548 (14)	[56]
**18b**	48 h: HeLa (7.5), MCF-7(16), A-548 (9.5)	[56]
**18c**	48 h: HeLa (2.2), MCF-7(1.0), A-548 (1.0)	[56]
**18d**	48 h: HeLa (7.7), MCF-7(13), A-548 (9.9)	[56]
**18e**	48 h: HeLa (3.1), MCF-7(7.4), A-548 (7.1)	[56]
**18f**	48 h: HeLa (5.2), MCF-7(9.6), A-548 (7.8)	[56]
**19a**	72 h: MCF-7 (2.15 ± 0.04), BxPC3 (2.46 ± 0.22), AsPC1 (2.29 ± 0.19), HuH7 (1.44 ± 0.05)	[57]
**19b**	72 h: MCF-7 (2.52 ± 0.12), BxPC3 (2.23 ± 0.60), AsPC1 (1.95 ± 0.10), HuH7 (1.43 ± 0.08)	[57]
**20a**	24 h: A-549 (>12.5), HCT 116 (11.30 ± 0.86), HeLa (7.30 ± 0.59), MDA-MB-231 (8.14 ± 0.02), SHSY5Y (>12.50), HASMC1 (10.81 ± 0.65), HASMC2 (6.31 ± 0.21). 72 h: A-549 (3.58 ± 0.67), HCT 116 (3.02 ± 1.11), HeLa (1.86 ± 1.34), MDA-MB-231 (3.05 ± 0.76), SHSY5Y (0.86 ± 0.99), HASMC1 (7.17 ± 0.13), HASMC2 (2.47 ± 0.32)	[58]
**20b**	24 h: A-549 (>12.5), HCT 116 (11.87 ± 0.66), HeLa (10.80 ± 1.63), MDA-MB-231 (>12.50), SHSY5Y (>12.50), HASMC1 (>12.5), HASMC2 (>12.5). 72 h: A-549 (1.93 ± 1.56), HCT 116 (1.79 ± 0.43), HeLa (3.13 ± 0.51), MDA-MB-231 (3.60 ± 0.37), SHSY5Y (1.08 ± 0.63), HASMC1 (>12.5), HASMC2 (>12.5)	[58]
**20c**	24 h: A-549 (>12.5), HCT 116 (>12.5), HeLa (9.16 ± 1.38), MDA-MB-231 (10.19 ± 0.49), SHSY5Y (>12.50), HASMC1 (>12.5), HASMC2 (7.42 ± 0.58). 72 h: A-549 (3.32 ± 0.40), HCT 116 (3.84 ± 0.10), HeLa (3.21 ± 0.53), MDA-MB-231 (3.70 ± 0.37), SHSY5Y (1.66 ± 0.25), HASMC1 (>12.5), HASMC2 (6.18 ± 0.34)	[58]
**21a**	24 h, dark: HeLa (9.57 ± 0.1), MCF-7 (4.78 ± 0.09). 24 h, visible light: HeLa (4.74 ± 0.1), MCF-7 (2.02 ± 0.07)	[59]
**21b**	24 h, dark: HeLa (24.45 ± 0.3), MCF-7 (>20). 24 h, visible light: HeLa (10.23 ± 0.3), MCF-7 (14.18 ± 0.1)	[59]
**21c**	24 h, dark: HeLa (8.95 ± 0.2), MCF-7 (2.99 ± 0.08). 24 h, visible light: HeLa (1.29 ± 0.04), MCF-7 (0.65 ± 0.03)	[59]
**21d**	24 h, dark: HeLa (8.80 ± 0.3), MCF-7 (8.74 ± 0.1). 24 h, visible light: HeLa (4.79 ± 0.2), MCF-7 (8.26 ± 0.2)	[59]
**21e**	24 h, dark: HeLa (6.10 ± 0.1), MCF-7 (4.13 ± 0.1). 24 h, visible light: HeLa (4.27 ± 0.1), MCF-7 (2.08 ± 0.09)	[59]
**22a**	72 h: PC3 (5.4), HL-60 (3.6), L02 (4.8)	[60]
**22b**	72 h: PC3 (3.1), HL-60 (2.4), L02 (3.4)	[60]
**23a**	72 h: HMLER (0.21 ± 0.01), HMLER-shEcad (0.32 ± 0.02), MCF10A (0.51 ± 0.01). 120 h: Mammosphere (0.54 ± 0.01)	[61]
**23b**	72 h: HMLER (0.22 ± 0.01), HMLER-shEcad (0.25 ± 0.01), 120 h: Mammosphere (1.26 ± 0.04)	[61]
**23c**	24 h: MCF-7 (1.6 ± 0.8), MDA-MB-231 (1.9 ± 1.2). 48 h: MCF-7 (1.2 ± 0.8), MDA-MB-231 (1.0 ± 0.9)	[62]
**24**	72 h: A-549 (4.2 ± 0.8), PC-3 (3.2 ± 0.2), MRC-5 (5.1 ± 0.3)	[63]
**25**	24 h: A-549 (5.26), A-431 (5.41), L132 (7.47)	[64]

**Table 6:** IC_50_ values of compounds **26–28b**.

Compound	Cell Line (IC_50_, µM)	Reference
**26**	24 h: HT29 (9.610), SW480 (11.30), SW620 (31.00). 96 h: HT29 (<0.001), SW480 (0.220), SW620 (1.220), HaCaT (0.719)	[65]
**27a**	24 h: A-549 (4.34), MCF-7 (6.50), HaCaT (11.19)	[66]
**27b**	24 h: A-549 (8.46), MCF-7 (8.68), HaCaT (16.01)	[66]
**28**	48 h: MCF-7 (3.227 ± 0.052), HepG2 (3.532 ± 0.055), A-549 (3.984 ± 0.185), 4T1 (3.311 ± 0.100), COS-7 (6.319 ± 0.022)	[67]

## Data Availability

Not applicable.

## Abbreviations

apaf1	apoptotic protease activating factor-1
ATOX-1	antioxidant 1 copper chaperone
Bak	Bcl-2 homologous antagonist/killer
Bax	Bcl-2 associated X protein
Bip	binding immunoglobulin protein (also known as GRP-78)
CCS1	superoxide dismutase 1 copper chaperone
ct-DNA	calf-thymus DNA
COX17	cytochrome c oxidase copper chaperone
DDIT3	DNA damage inducible transcript 3
DNA Topo1	DNA topoisomerase 1
DPQ	dipyridoquinoxaline
DPPZ	dipyridophenazine
GSH	glutathione
γ-H2AX	phosphorylated H2A histone family member X
hCtr1	human Copper transporter 1
IC50	drug concentration required for 50% inhibition of cell growth
IRE1	inositol-requiring enzyme 1
MMP-2	matrix metalloprotease-2
NSAID	nonsteroidal anti-inflammatory drug
p53	tumour protein p53
PARP	poly (ADP-ribose) polymerase
PERK	protein kinase R (PKR)-like endoplasmic reticulum kinase
SOD	superoxide dismutase
VEGFR-1	vascular endothelial growth factor receptor 1
